# Interplay between Oxo and Fluoro in Vanadium Oxyfluorides for Centrosymmetric and Non-Centrosymmetric Structure Formation

**DOI:** 10.3390/molecules26030603

**Published:** 2021-01-24

**Authors:** Prashanth Sandineni, Hooman Yaghoobnejad Asl, Weiguo Zhang, P. Shiv Halasyamani, Kartik Ghosh, Amitava Choudhury

**Affiliations:** 1 Department of Chemistry, Missouri University of Science and Technology, Rolla, MO 65409, USA; sandinenip@mst.edu (P.S.); hynr8@mst.edu (H.Y.A.); 2 Department of Chemistry, University of Houston, 112 Fleming Building, Houston, TX 77204, USA; wgzhang82@gmail.com (W.Z.); pshiv@Central.UH.EDU (P.S.H.); 3 Department of Physics, Astronomy and Materials Sci, Missouri State University, 901 S. National Ave., Springfield, MO 65897, USA; KartikGhosh@MissouriState.edu

**Keywords:** vanadium oxy-fluoride, non-centrosymmetric crystal, second-harmonic generation

## Abstract

Herein, we report the syntheses of two lithium-vanadium oxide-fluoride compounds crystallized from the same reaction mixture through a time variation experiment. A low temperature hydrothermal route employing a viscous paste of V_2_O_5_, oxalic acid, LiF, and HF allowed the crystallization of one metastable phase initially, Li_2_VO_0.55_(H_2_O)_0.45_F_5_⋅2H_2_O (**I**), which on prolonged heating transforms to a chemically similar yet structurally different phase, Li_3_VOF_5_ (**II**). Compound **I** crystallizes in centrosymmetric space group, *I*2/*a* with *a* = 6.052(3), *b* = 7.928(4), *c* = 12.461(6) Å, and *β* = 103.99(2)°, while compound **II** crystallizes in a non-centrosymmetric (NCS) space group, *Pna*2_1_ with *a* = 5.1173(2), *b* = 8.612(3), *c* = 9.346(3) Å. Synthesis of NCS crystals are highly sought after in solid-state chemistry for their second-harmonic-generation (SHG) response and compound **II** exhibits SHG activity albeit non-phase-matchable. In this article, we also describe their magnetic properties which helped in unambiguous assignment of mixed valency of V (+4/+5) for Li_2_VO_0.55_(H_2_O)_0.45_F_5_⋅2H_2_O (**I**) and +4 valency of V for Li_3_VOF_5_ (**II**).

## 1. Introduction

There has been a surge of research activities in the area of heteroanionic materials such as oxychalcogenides, oxynitrides, oxypnictides, and oxyhalides [1]. By virtue of the difference in charges, electronegativity, and sizes these heteroanions modulate the electronic properties and induce structural complexities in the material [2,3,4,5]. Often heteroanionic materials are more interesting than the pure homoanionic oxides, especially in the case of compounds with transition metals, where the presence of heteroanions influence the iono-covalency of bonds and oxidation state of the transition metal, often producing mixed valency [6,7]. Tuning of iono-covalency through fluoride substitution on oxy-site has been fruitful in tuning Li-ion battery voltage as exemplified by LiFePO_4_(OH)_(1-x)_F_x_ [8] and Na_3_V_2_(PO_4_)_2_O_2_F-Na_3_V_2_(PO_4_)_2_F_3_ [9,10] systems. The heteroanion also induce acentric polyhedra, the parallel and anti-parallel alignment of which can lead to non-centrosymmetric and symmetric crystal system, respectively [11,12]. Synthesis of the heteroanionic solid is also challenging because of the different energetics of the metal-heteroanion bond, let alone the problem of mixed occupancy/disorder [13,14]. One of the important systems of heteroanionic compounds that has been explored to some extent relates to vanadium oxy-fluorides, which often contain a V = O terminal bond, especially at +4/+5 oxidation state, that can be exchanged with a fluoride under appropriate conditions [15,16,17,18,19]. The Poeppelmeir group has investigated this system in presence of sodium and other ions, which has resulted in materials that show interesting electrochemistry and second-harmonic-generation (SHG) activities, respectively [17,18,19]. Synthesis of the latter producing the non-centrosymmetric (NCS) crystals also exemplifies how the same reactant composition can lead to two different NCS crystals with varying amounts of fluorine as a function of time and temperature [19]. Inspired by this work we wanted to investigate the Li-V-O-F system under the same temperature and reactant composition but as a function of time. This investigation has led to the discovery of two new Li-V-oxy-fluoride phases with centrosymmetric and non-centrosymmetric structures of the compositions, Li_2_VO_0.55_(H_2_O)_0.45_F_5_⋅2H_2_O (**I**) and Li_3_VOF_5_ (**II**), through the interplay of fluorine-oxy ratio. In this manuscript, we present the synthesis, crystals structures, and magnetic properties of the two materials and SHG activity of the latter.

## 2. Results and Discussion

### 2.1. Synthesis

**Figure 1:**
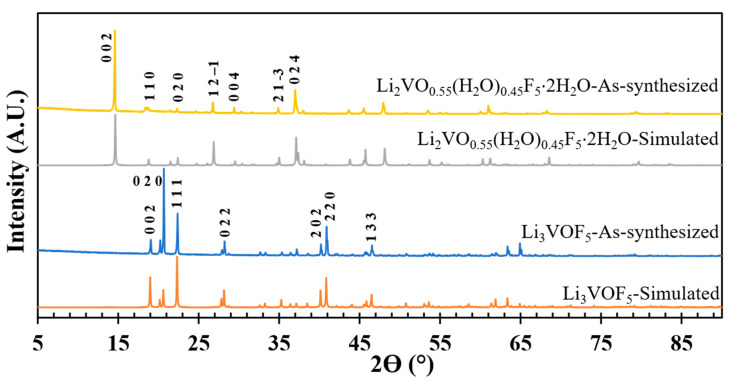
Observed and simulated powder X-ray diffraction patterns of Li_2_VO_0.55_(H_2_O)_0.45_F_5_⋅2H_2_O **(I)** and Li_3_VOF_5_ **(II)**.

In this article we are reporting syntheses of two compounds, Li_2_VO_0.55_(H_2_O)_0.45_F_5_⋅2H_2_O (**I**) and Li_3_VOF_5_ (**II**), that originate from the same reaction mixture. Our initial efforts to synthesize oxide-fluoride phases in the Li-V-O-F system failed when the reactions were carried out in hydrothermal conditions in the presence of hydrofluoric acid (HF), due to the formation of full-fluoro Li_3_VF_6_ (discussed in Materials and Methods). To avoid the formation of the full-fluoro phase, we avoided excessive amounts of water in the reaction. The reaction was carried out from a mixture of flux-like paste consisting of V_2_O_5_, C_2_O_4_H_2_⋅2H_2_O (oxalic acid dihydrate) and LiF in the presence of 1 ml HF in hydrothermal autoclave (details are given in Materials and Methods section). The oxalic acid is a known reducing agent for the penta-valent vanadium oxide precursor. The same ratio of reactants was allowed to undergo hydrothermal reactions and eventually on the sixth day the desired NCS oxide fluoride compound, Li_3_VOF_5_ (**II**), formed after transformation from a metastable phase in the course of reaction. The first structure that immediately formed after one day, Li_2_VO_0.55_(H_2_O)_0.45_F_5_⋅2H_2_O (**I**), was a layered structure, which on dimension reduction [20] over time transformed into isolated [VOF_5_]^3–^ acentric octahedral unit through the replacement of one of the O/H_2_O by terminal full ‘= O’ bond. Crystal structures and phase compositions for both the compounds were determined by single-crystal X-ray diffraction (details in Materials and Methods). Crystallographic parameters and refinement data for **I** and **II** are given in Table 1. Phase purity of the samples was evaluated by laboratory PXRD through the direct comparison with the simulated pattern generated from the atomic coordinates of single-crystal XRD solution (Figure 1). The PXRDs match very well with the simulated patterns indicating that the as-synthesized phases are mostly pure.

### 2.2. Structure Description

**Figure 2:**
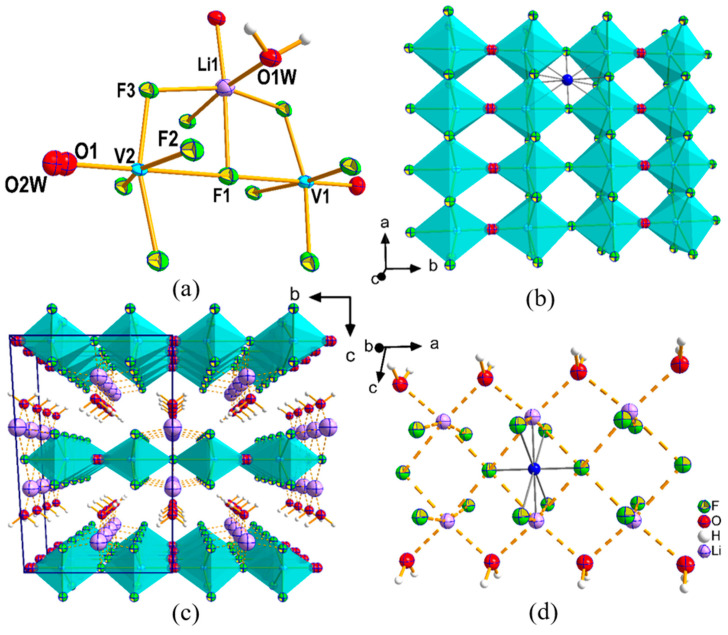
(**a**) Coordination of different atoms in **I** is shown. Only atoms that appeared in asymmetric unit are labeled. Thermal ellipsoids are given at 50% probability. (**b**) The center of inversion within the ribbon is shown with a blue dummy atom. V-octahedra are shown with cyan color. (**c**) Stacking of layers showing the presence of Li-ions and water in the inter-layer space. (**d**) The center of inversion within the Li-centric polyhedral layers is shown with blue dummy atom.

One of the oxy-fluorides, **I**, crystallizes in centrosymmetric *I*2/*a* space group while the other (**II**) in non-centrosymmetric space group, *Pna*2_1_. The asymmetric unit of **I** contains eight non-hydrogen atoms consisting of two vanadium, three fluorine, one disordered terminal oxygen/water, one lithium, and one oxygen from the water molecule (Figure 2a). Both V sites have partial occupancies with 32 and 68% for V1 and V2, respectively. Both V atoms form octahedral coordination surrounded by 5 F and one disordered O1/H_2_O(O2w). V1 and V2 acentric octahedra are connected to each other through corner-sharing via F1 and such dimers are again edge-shared (via F2 and F3) between themselves to create a ribbon. In the ribbon, V1 and V2 octahedra alternate in such way that their connections also create inversion centers within the ribbon as shown in Figure 2b. Such ribbons, though apparently connected through a disordered O(1)/H_2_O(O2w) but in reality, disordered atoms are either terminal V = O from one vanadium atom or terminal V—H_2_O from the other vanadium as the sum of the two V atoms make up to one. Such disorder has been previously found in a Na-V-oxy-fluoride [19]. Note that each of these V-octahedra are acentric but their anti-parallel arrangements create the center of inversions. These layers/ribbons are stacked along the *c*-axis and the cohesion between them is ascertained by the presence of lithium ions, where each Li-ion adopts octahedral coordination with four F atoms and two water molecules (Figure 2c). Here again, Li is off-centric in the octahedron but the juxtaposition of two such acentric octahedron creates a center of inversion (Figure 2d). All the important inter-atomic distances in **I** are listed in Table S1 (SI), and a figure showing all the symmetry elements present in **I** is also given in SI (Figure S1).

**Figure 3:**
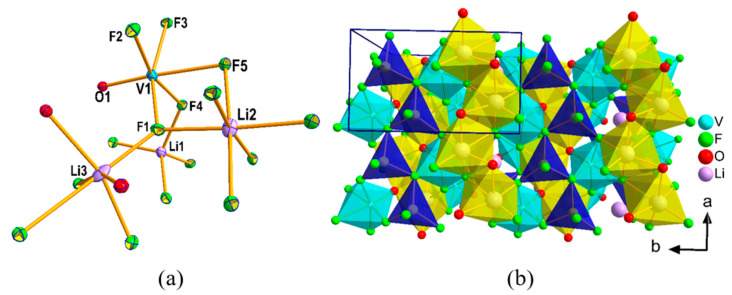
(**a**) Coordination of different atoms in **II** is shown. Only atoms that appeared in asymmetric unit are labeled. Thermal ellipsoids are given at 50% probability. (**b**) The arrangement of acentric polyhedra, Li(1)F_4_ (Blue), Li(3)O_2_F_4_ (yellow) and V(1)OF_5_ (cyan) are shown to be pointing in same direction.

The asymmetric unit of **II** contains ten atoms with five fluorine, one oxygen, one vanadium, and three lithium atoms (Figure 3a). Unlike **I**, all atoms of **II** are fully occupied and there is no disorder in any atom. The structure is built up of two Li-centered and one V-centered octahedral building units and one Li-centered tetrahedral unit. V1 is surrounded by 5 F and one oxygen, which has a short metal—O distance (1.664 Å) indicating V = O bond. Li3 is surrounded by four F and two O, while Li1 and Li2 are exclusively surrounded by F atoms in four and six coordination, respectively. All the important interatomic distances in **II** are listed in Table S2. V1 and Li3 in V(1)OF_5_ and Li(3)F_4_O_2_ octahedral units, respectively, are off-centric. In this structure, VOF_5_ octahedra are isolated, meaning they are not connected to each other through a bridging F or O and are held together by ionic forces between the [VOF_5_]^3−^ and the Li-ions. In this structure, the arrangement of acentric polyhedral units is such that they lack a center of symmetry. For example, all the Li(1)F_4_ tetrahedra point in one direction, similarly all the V(1)OF_5_ and Li(3)O_2_F_4_ are also parallel (Figure 3b). Such arrangements of acentric polyhedra ultimately lead to the crystallization in the NCS space group and thus, become a candidate for second harmonic generation (SHG) material. It is also worthwhile to discuss here that the structure of **II** is similar to the alpha form of Li_3_AlF_6_ [21]. There is a large variety of structures related to the mineral cryolite, Na_3_AlF_6_, that can be found with various alkali metals (or mixed alkali metals) with main-group or transition metal fluorides, A_3_MF_6_ (A = Li, Na, K, Li/K, Li/K, Li/Na; M = Al, Ga, Ti, V, Cr, Fe) [21,22,23,24]. These compounds are known to display phase-transition from an idealized cubic structure (high temperature form) to a monoclinic (beta-form) or orthorhombic (alpha-form) crystal system based on the ability of alkali-ions to accommodate different coordination. The orthorhombic, alpha-form crystallizes in the non-centrosymmetric space group, *Pna*2_1_, and almost all the pure Li-form of Li_3_MF_6_ (M = Al, Ga, Ti, V, Cr, Fe) are known to display $\alpha \underset{T}{↔}\beta \;$ transition as a function of temperature [24]. However, there exist subtle differences between the current Li_3_VOF_5_ (**II**) and the Li_3_AlF_6_ [21] or Li_3_VF_6_ [25]. In α- Li_3_MF_6_ (M = Al and V), all the Li-ions adopt ideal/distorted octahedral coordination but in **II** one of the Li-ions adopts a perfect tetrahedral coordination. Another significant difference lies in the distortion of MF_6_ (M = Al and V) octahedra in α- Li_3_MF_6_; in **II**, VOF_5_ octahedron is very distorted with totally off-center location of V while minimum distortion is observed in MF_6_ (M = Al and V) octahedron of α- Li_3_MF_6_ (M = Al and V). Besides these structural differences brought about by the replacement of one F by a terminal, double bonded oxygen (V = O) in α- Li_3_VF_6_, a major change is also resulted in the oxidation state of V, which is +4 in **II** but +3 in Li_3_MF_6_ (M = Al, Ga, Ti, V, Cr, Fe). Since metal oxygen double bond, M = O is prevalent only in few metals such as V, Mo, and Nb, this oxy-fluoride structure is not expected to be found in other metals for which Li_3_MF_6_ (M = Al, Ga, Fe, Cr) is quite common. The NCS α-form of Li_3_VF_6_ has been synthesized from the rapid cooling of melt (mixture of LiF and VF_3_), while the NCS form of oxy-fluoride can be achieved under mild hydrothermal conditions under prolonged reaction time (six days).

### 2.3. IR Spectra

**Figure 4:**
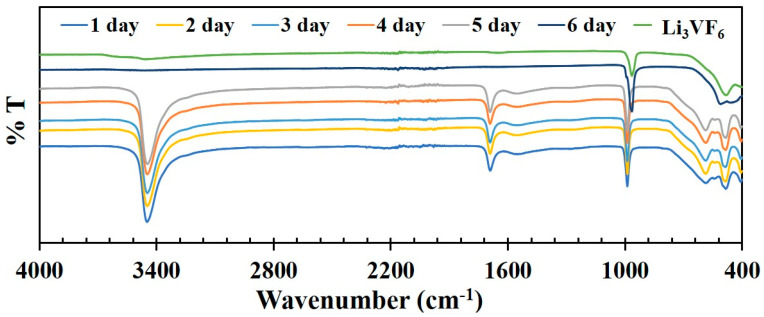
FT-IR spectra of the compounds synthesized from 1 to 6 days.

Figure 4 shows the FT-IR spectra of the compounds synthesized within 1 to 6 days, explaining the progression of reaction from Li_2_VO_0.55_(H_2_O)_0.45_F_5_⋅2H_2_O (**I**) in 1 day to Li_3_VOF_5_ (**II**) in 6 days. The bands near 3440 and 1680 cm^–1^ correspond to O-H stretching and bending modes due to water molecules present in Li_2_VO_0.55_(H_2_O)_0.45_F_5_⋅2H_2_O (**I**) synthesized from 1 to 5 days reactions. These bands disappeared completely in the sixth day reaction product, which also confirms that there is no water or OH groups present in the compound as confirmed by the X-ray determined structure, Li_3_VOF_5_ (**II**). The band around 987 cm^−1^ corresponds to V = O stretching, which is present in the compound Li_2_VO_0.55_(H_2_O)_0.45_F_5_⋅2H_2_O (**I**) (1–5 days product) as well as in Li_3_VOF_5_ (**II**) (6 days duration reaction) where it appears as a shoulder, followed by a broad peak at 963 cm^–1^ which is due to V–F stretching and matches well with the IR spectra of beta-Li_3_VF_6_ (Li_3_VF_6_ has been obtained from an independent synthesis described in Materials and Methods).

### 2.4. Magnetic Measurements

**Figure 5:**
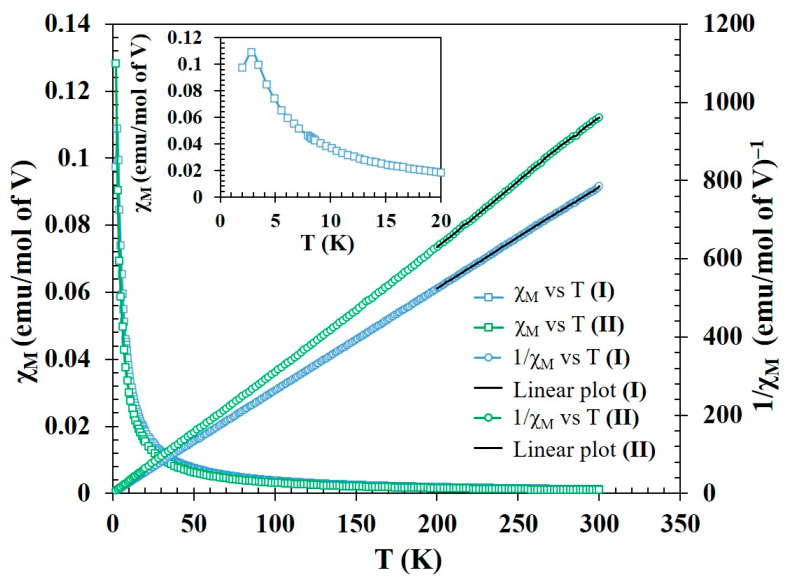
Temperature dependence of molar magnetic susceptibility (χ_M_) and inverse molar magnetic susceptibility (χ_M_^–1^) of Li_2_VO_0.55_(H_2_O)_0.45_F_5_⋅2H_2_O (**I**) and Li_3_VOF_5_ **(II)**. (The inset shows the onset of anti-ferromagnetic ordering in **II**).

The temperature dependent ZFC magnetic susceptibility, χ_M_(T), and the corresponding inverse molar susceptibility, χ_M_^−1^(T), for the as-synthesized compounds are given in Figure 5. The magnetic susceptibility χ_M_ vs. T plot for Li_2_VO_0.55_(H_2_O)_0.45_F_5_⋅2H_2_O (**I**) at an applied field of 1 T is asymptotic in the temperature range 2 to 300 K, indicating paramagnetic interactions without any ordering. The positive Θp value (12.8 K) suggests that there exists a ferromagnetic correlation in the paramagnetic region, which can lead to an ordering at further low temperatures below 2 K (not measured).

However, in the case of Li_3_VOF_5_, there is a kink at 2.84 K (inset of Figure 5), corresponding to the onset of anti-ferromagnetic ordering. Magnetic moment/V as calculated from the curie constant, C, derived from the linear fit of the χ_M_^−1^ vs. T data in the temperature range 200–300 K yields a value of 1.54 μB/V for **I**, which is less than the theoretical magnetic moment of one unpaired *d*-electron (*d*^1^) as it should be in the case of pure V^4+^. This lower magnetic moment accounts for 89% of theoretical moment of a *d*^1^ system (1.73 μB), which also reinforces that V is in mixed oxidation states of +4 and +5. Therefore, oxidation state of V can be split as 0.89 V^4+^ and 0.11 V^5+^, which establishes an overall oxidation state of +4.11/V and creates a charge-balanced formula for Li_2_VO_0.55_(H_2_O)_0.45_F_5_⋅2H_2_O (**I**). On the other hand, similar calculations for compound **II** yields a magnetic moment of 1.75 μB/V. This value confirms that V in Li_3_VOF_5_ is in +4 oxidation state, which matches well with the theoretical moment of the *d*^1^ (1.73 μB) system. The isothermal magnetization plot (*M* vs. *H*) of **I** at 5 K shows that the saturation magnetization is about 0.46 N*β* at the highest applied field of 5 T. This value is about 50% of the saturation magnetization considering ferromagnetic alignment of spins (Figure S2).

### 2.5. SHG Measurements

**Figure 6:**
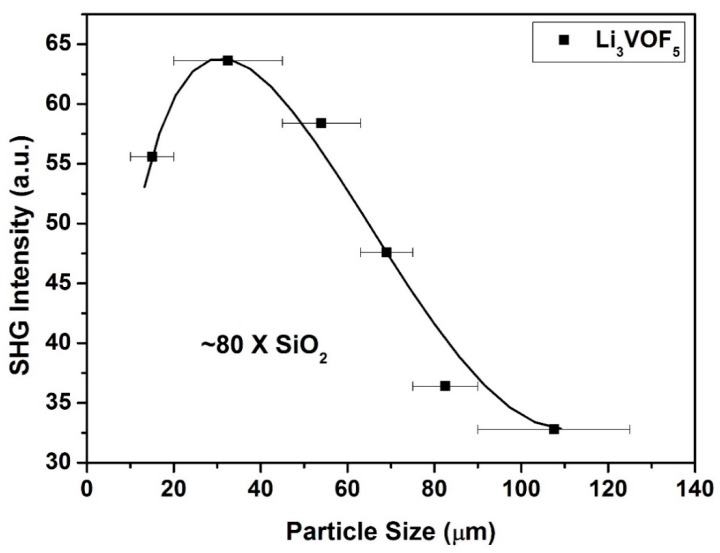
Second-harmonic-generation (SHG) measurements of the compound Li_3_VOF_5_ (**II**).

As the powder SHG efficiency has been shown to depend strongly on particle size, Li_3_VOF_5_ was sieved into distinct particle size ranges (<20, 20–45, 45–63, 63–75, 75–90, 90–125 μm) to investigate its phase-matching behavior. Polycrystalline BaTiO_3_ was also sieved into similar particle sizes for SHG efficiency comparison. BaTiO_3_ is used as standard sample for non-phase matching materials and the intensity of BaTiO_3_ is taken 400 times larger than that of alpha-SiO_2_ [26]. The efficiency of sample is calculated by the equation, efficiency = I sample/I BaTiO_3_*400 = 63.6/332*400 = 76.6~80. Figure 6 shows that Li_3_VOF_5_ has around 80 times larger SHG intensity than that of alpha- SiO_2_. However, the phase behavior is not phase-matchable. For industrial application, phase-matchable SHG materials are more desirable. Non-phase-matchable behavior has been observed previously in a V-oxy-fluoride compound containing Na-ions, NaVOF_4_(H_2_O), for which SHG response was similar to α- SiO_2_ [19]. However, in Li_3_VOF_5_, SHG response is 80 times larger than α- SiO_2_, which may be due to polar alignments of a greater number of acentric polyhedra. However, for organic molecular NCS crystals, phase-matchability behavior has been understood quite well [27,28], in case of solid-state inorganic structures, rules governing the phase-matchability SHG response is not fully understood.

## 3. Materials and Methods

### 3.1. Caution

Hydrofluoric acid is toxic and corrosive and must be handled with extreme caution and the appropriate protective gear!

### 3.2. Materials

All the chemicals used in the syntheses were as purchased and without further purification. V_2_O_5_ (99.6%) was purchased from Aldrich. Oxalic acid dihydrate and LiF were purchased from Fisher Scientific and HF from Acros Organics.

### 3.3. Synthesis

Li_3_VOF_5_ (**II**) was synthesized by taking a mixture of 0.455 g of V_2_O_5_ (2.5 mmol), 0.3152 g of oxalic acid dihydrate (2.5 mmol), 0.2594 g of LiF (10 mmol) and 1 mL of HF in a 23 mL capacity Teflon-lined stainless steel Paar acid digestion bomb. The paste-like mixture was formed inside the Teflon cup, which was then covered with a lid, placed in the steel autoclave, sealed tightly, and placed in a 185 °C pre-heated oven. The autoclave was heated for 6 days, followed by cooling down naturally to room temperature. The product of the reaction consisted of green colored crystals, which was filtered and washed with hot water. A good quality crystal was selected for single-crystal X-ray diffraction study. The bulk product was then finely ground into fine powder for laboratory X-ray diffraction.

In order to observe the reaction progress, time-dependent reactions were carried out in which reaction products were monitored by ex situ PXRD and IR spectra from reactions spanning one to seven days duration. When the reaction was carried out for one day, turquoise colored crystals were formed which appeared different from the Li_3_VOF_5_ phase. Single-crystal X-ray diffraction study revealed the composition to be Li_2_VO_0.55_(H_2_O)_0.45_F_5_⋅2H_2_O (**I**). As the time increased from one day to longer duration, the compound Li_2_VO_0.55_(H_2_O)_0.45_F_5_⋅2H_2_O (**I**) continued forming until the fifth day. On the sixth day there was an emergence of another phase, Li_3_VOF_5_ (**II**), as confirmed by the PXRD, which did not transform to any other phase on the seventh day.

As mentioned earlier, that formation of Li_3_VF_6_ was ubiquitous when excess water was used as in standard hydrothermal synthesis. Li_3_VF_6_ can be synthesized by taking a mixture of 0.455 g of V_2_O_5_ (2.5 mmol), 0.6304 g of oxalic acid dihydrate (5 mmol), 0.3891 g of LiF (15 mmol), and 0.6 mL of HF (15 mmol) added in 5 mL of water in a 23 mL capacity Teflon-lined stainless steel Paar acid digestion bomb and placed in a 185 °C pre-heated oven. The autoclave was heated for 2 days, followed by cooling down naturally to room temperature. The product of the reaction consisted of green colored crystals, which was filtered and washed with hot water. This product was used to compare the IR spectra.

## 4. Material Characterization

### 4.1. Single-Crystal X-ray Diffraction

Single-crystal X-ray diffraction data were collected from crystals obtained from the syntheses of one (**I**) and six (**II**) days reactions. The intensity data sets were collected on a Bruker Smart Apex diffractometer equipped with monochromated Mo Kα radiation (0.7107 Å). The data sets were collected at 200 K for **I** and at room temperature for **II** using Apex II or SMART [29] software employing a scan of 0.3° in ω with an exposure time of 20 s per frame. The cell refinement and data reduction were carried out with SAINT [30] and the program SADABS [30] was used for the absorption correction. The structures were solved by direct methods using SHELX-97 and difference Fourier syntheses [31]. Full-matrix least-squares refinement against |F^2^| was carried out using the SHELXTL-PLUS suite of programs [30]. Finally, all the atoms were refined anisotropically using SHELXL 2018 incorporated in SHELXLe [32]. Details of final refinements and cell parameters of the compounds are given in Table 1. Selected bond lengths are given in Tables S1 and S2. Final atomic coordinates and isotropic displacement parameters are given in Tables S3 and S4.

### 4.2. Powder X-ray Diffraction (PXRD)

The laboratory PXRD patterns were obtained from a PANalytical X’Pert Pro diffractometer equipped with a Cu Kα1,2 anode and a linear array PIXcel detector over a 2θ range of 5 to 90° with an average scanning rate of 0.0472°s^−1^. Figure S3 represents the PXRDs showing progression of the reaction from Li_2_VO_0.55_(H_2_O)_0.45_F_5_⋅2H_2_O (**I**) (1 day) to Li_3_VOF_5_ (**II**) (6 and 7 days). The initial structure is stable until 5 days of reaction duration.

### 4.3. IR Spectroscopy

The IR spectrum was collected using Thermo Nicolet iS50 FT-IR spectrometer over 400–4000 cm^−1^ on a sample using ATR mode.

### 4.4. Magnetic Measurements

The variable temperature magnetic susceptibility measurements of the compounds (as-prepared) were carried out in the temperature range 2–300 K using Quantum Design SQUID magnetometer at 1T applied field while warming up under field cooled (FC) and zero-field cooled (ZFC) conditions.

### 4.5. SHG Measurements

Powder SHG measurements were performed on a modified Kurtz-NLO system using a pulse Nd:YAG laser (Quantel Laser, Ultra 50) with a wavelength of 1064 nm.

## 5. Conclusions

The centrosymmetric material, Li_2_VO_0.55_(H_2_O)_0.45_F_5_⋅2H_2_O (**I**), has a layered structure stabilized by hydrated Li-ion in the inter-layer space. On the other hand, the NCS material, Li_3_VOF_5_ (**II**), contains a distorted and isolated [VOF_5_]^3–^ octahedral unit. In Li_2_VO_0.55_(H_2_O)_0.45_F_5_⋅2H_2_O (**I**), two vanadium atoms are partially occupied and together their occupancies add up to one and the average oxidation state of V is +4.11. By increasing the synthesis time further, reduction of dimension and V oxidation state take place to generate a perfectly ordered NCS phase, Li_3_VOF_5_ (**II**) with V in +4 oxidation state. NCS phase in vanadium is interesting due to the presence of unpaired electrons, which can lead to multiferroic materials. Compound **II** is SHG-active material with non-phase-matchable characteristics, which adds to the list of other non-phase-matchable SHG materials in inorganic solids and will aid in enhancing our understanding of future synthesis of targeted SHG-active material.

## Figures and Tables

**Table 1:** Crystal data and structure refinement for Li_2_VO_0.55_(H_2_O)_0.45_F_5_⋅2H_2_O (**I**) and Li_3_VOF_5_ (**II**).

Empirical Formula	Li_2_VO_0.55_(H_2_O)_0.45_F_5_⋅2H_2_O	Li_3_VOF_5_
Formula weight	212.76	182.76
Temperature	200 (2) K	298 (2) K
Wavelength	0.71073 Å	0.71073 Å
Crystal system	Monoclinic	Orthorhombic
Space group	*I*2/*a*	*Pna*2_1_
*a*	6.052(3) Å	5.1173(2) Å
*b*	7.928(4) Å	8.612(3) Å
*c*	12.461(6) Å	9.346(3) Å
*α*	90°	90°
*β*	103.99(2)°	90°
*γ*	90°	90°
Volume	580.1 (5) Å^3^	411.9 (2) Å^3^
Z	4	4
Density (calculated)	2.436 g/cm^3^	2.947 g/cm^3^
F(000)	412	340
Reflections collected	10964	3537
Independent reflections	1273 [R(int) = 0.0966]	770 [R(int) = 0.0414]
Goodness-of-fit on F^2^	1.033	1.059
Final R indices [I > 2sigma(I)]	R_1_ = 0.0668, wR_2_ = 0.1733	R_1_ = 0.0286, wR_2_ = 0.0635
R indices (all data)	R_1_ = 0.0878, wR2 = 0.1955	R_1_ = 0.0329, wR_2_ = 0.0659
Largest diff. peak and hole	1.017 and −1.198 e·Å^−3^	0.415 and −0.345 e·Å^−3^

## Data Availability

Data can be obtained from the authors.

## Supplementary Materials

The following are available online. Tables of important bond lengths and atomic coordinates (Tables S1–S4); Figures showing symmetry elements of **I** (Figure S1), M-H plot of **I** (Figure S2), and Powder X-Ray diffraction patterns (Figure S3). CCDC No. 2053006 and 2053005 for crystal structures of **I** and **II**.

## Sample Availability

Samples of the compounds are available from the authors.
