# Supplementary material for: Interplay between Oxo and Fluoro in Vanadium Oxyfluorides for Centrosymmetric and Non-Centrosymmetric Structure Formation
Source: Molecules. 2021 Jan 24;26(3):603. doi: 10.3390/molecules26030603 (PMC7866034; doi:10.3390/molecules26030603)
Supplement: Supplementary file 1 [file molecules-26-00603-s001.pdf]

Interplay Between Oxo And Fluoro In Vanadium Oxyfluorides For Centrosymmetric And Non-Centrosymmetric Structure Formation

Prashanth Sandineni,[a] Hooman Yaghoobnejad Asl,[a] Weiguo Zhang,[b] P. Shiv Halasyamani,[b] Kartik Ghosh,[c] Amitava Choudhury[a]*

[a] Department of Chemistry, Missouri University of Science and Technology, Rolla, MO 65409, USA

[b] Department of Chemistry, University of Houston, 112 Fleming Building, Houston, TX 77204-5003, USA

[c] Department of Physics, Astronomy and Materials Sci, Missouri State University, 901 S. National Ave., Springfield, MO 65897, USA

* Correspondence: choudhurya@mst.edu

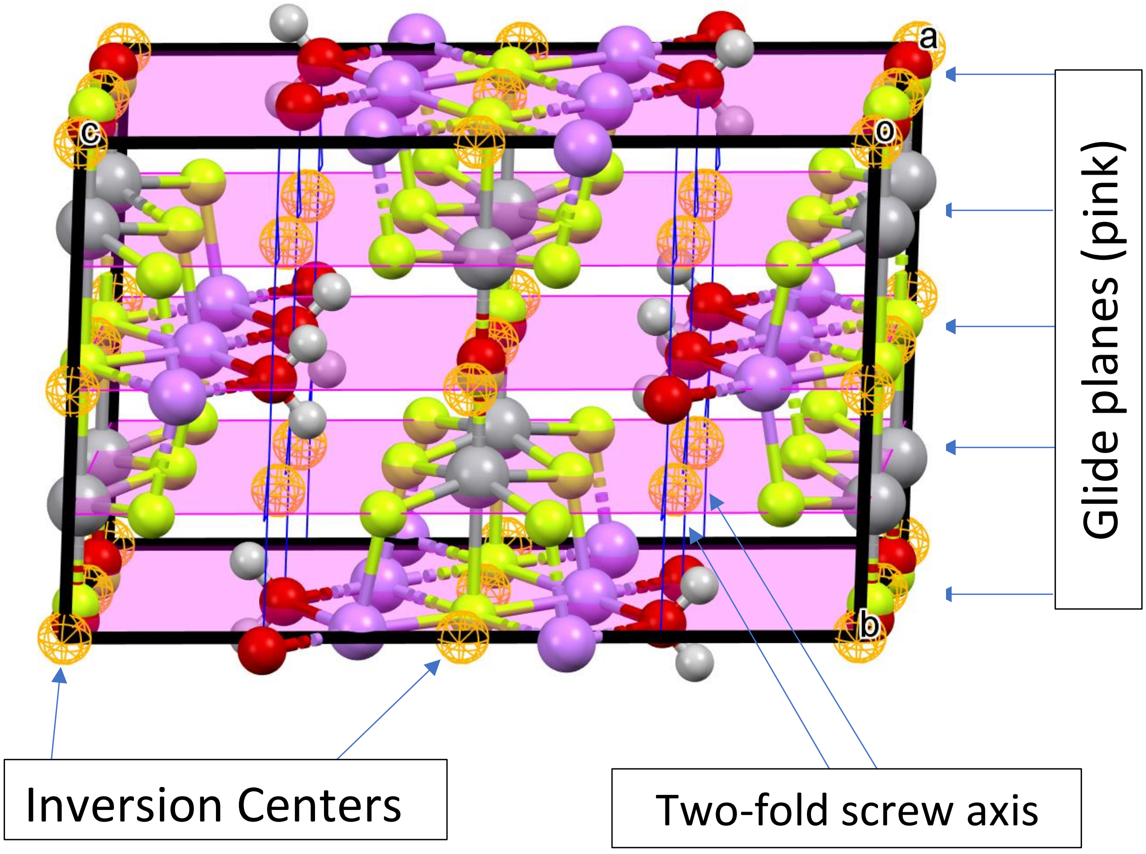

Figure S1. The structure of **I** showing the locations of different symmetry elements.

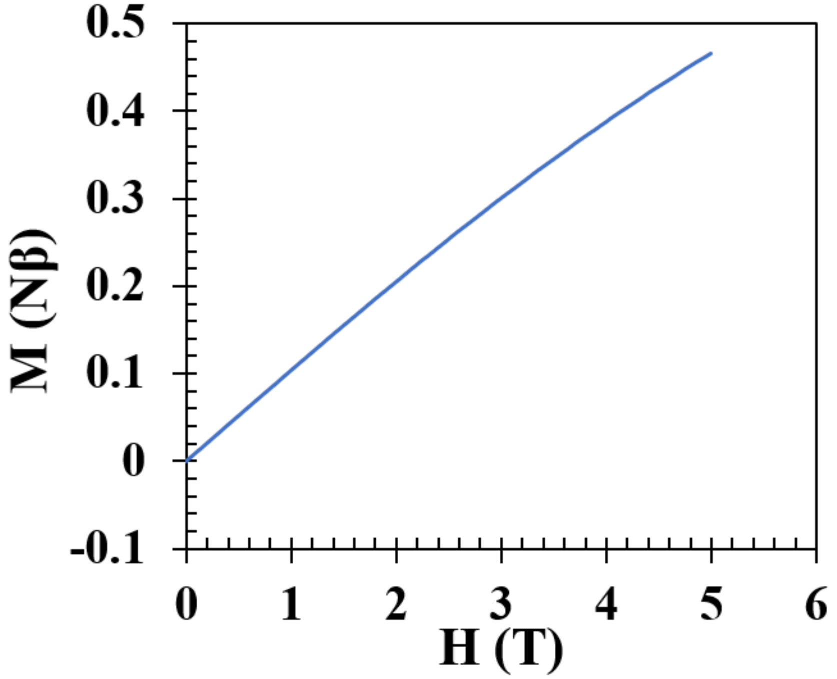

Figure S2. Isothermal M vs H plot at 5 K for $Li_2VO_{0.55}(H_2O)_{0.45}F_5{\cdot}2H_2O$ (**I**).

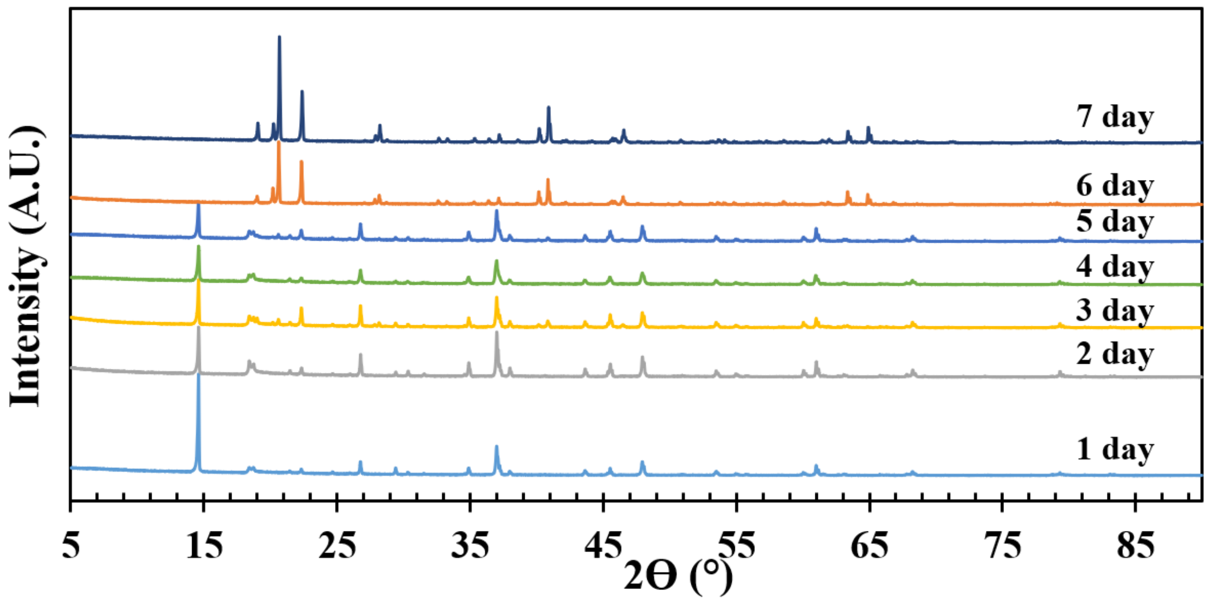

Figure S3. Comparison of PXRDs during the progression of reaction from 1 to 7 days.

Table S1. Selected interatomic distances [Å] for $Li_2VO_{0.55}(H_2O)_{0.45}F_5 \cdot 2H_2O$.

Atom-Atom	*d* (Å)	Atom-Atom	*d* (Å)
V1 – F1	2.234(3)	Li1 – F1	2.223(5) #6
2 x V1 – F2	2.043(17) #2	Li1 – F1	2.230(5) #4
2 x V1 – F3	2.046(18) #3	Li1 – F2	1.936(4) #5
V1 – O1/O2W	1.700(4) #1	Li1 – F3	1.940(4)
V1 – O1/O2W	1.930(3) #1	Li1 – O1W	2.011(5) #5
V2 – F1	2.196(2)	Li1 – O1W	2.013(6)
2 x V2 – F2	1.983(17)		
2 x V2 – F3	1.986(17)		
V2 – O1/O2W	1.800(4)		
V2 – O1/O2W	1.570(3)		

Symmetry transformations used to generate equivalent atoms:

#1 x,y+1,z #2 x-1/2,-y+1,z #3 -x+1,-y+1,-z

#4 -x,-y+1,-z #5 x+1/2,-y+1,z #6 -x+1/2,y,-z

Table S2. Selected interatomic distances [Å] for Li_3VOF_5.

Atom-Atom	*d* (Å)	Atom-Atom	*d* (Å)	Atom-Atom	*d* (Å)
V1 – O1	1.664(5)	Li1 – F2	1.858(17) #4	Li2 – F5	1.979(13) #3
V1 – F1	1.880(4)	Li1 – F2	1.874(13) #2	Li2 – F5	2.092(14)
V1 – F2	1.908(4)	Li1 – F3	1.870(16) #5	Li3 – F1	1.930(17)
V1 – F3	1.973(4)	Li1 – F4	1.857(16)	Li3 – F3	2.010(16) #2
V1 – F4	1.968(3)	Li2 – F1	1.954(13)	Li3 – F5	2.032(14) #2
V1 – F5	2.127(3)	Li2 – F3	2.059(14) #1	Li3 – F5	2.451(18) #6
		Li2 – F4	2.017(13) #3	Li3 – O1	2.086(17) #7
		Li2 – F4	2.084(14) #1	Li3 – O1	2.191(13) #1

Symmetry transformations used to generate equivalent atoms:

#1 x+1,y,z #2 -x+1,-y+1,z+1/2 #3 x+1/2,-y+1/2,z

#4 -x+3/2,y+1/2,z+1/2 #5 -x+2,-y+1,z+1/2

#6 -x+1/2,y-1/2,z+1/2 #7 x+1/2,-y+3/2,z

Table S3. Atomic Coordinates and Equivalent Isotropic Displacement Parameters for $Li_2VO_{0.55}(H_2O)_{0.45}F_5{\cdot}2H_2O$.

Atom	**Wyck.**	**Occupancy**	**x/a**	**y/b**	**z/c**	**U [Å^2]**
V1	4e	0.32	0.2500	0.7777(2)	0.0000	0.016(1)
V2	4e	0.68	0.2500	0.2189(1)	0.0000	0.012(1)
F1	4e	1	0.2500	0.4959(2)	0.0000	0.022(1)
F2	8f	1	0.5461(3)	0.2597(2)	0.1068(1)	0.022(1)
F3	8f	1	0.0604(3)	0.2591(2)	0.1073(1)	0.022(1)
O1	4e	0.55	0.2500	0.0210(11)	0.0000	0.023(3)
O2W	4e	0.45	0.2500	-0.0080(9)	0.0000	0.023(4)
O1W	8f	1	0.3725(3)	0.5023(2)	0.2447(2)	0.022(1)
H1	8f	1	0.3850(5)	0.5820(5)	0.2910(3)	0.031(1)
H2	8f	1	0.3910(6)	0.4720(5)	0.2880(3)	0.031(1)
Li1	8f	1	0.0680(9)	0.5000(5)	0.1350(4)	0.025(1)

Table S4. Atomic Coordinates and Equivalent Isotropic Displacement Parameters for Li_3VOF_5.

Atom	**Wyck.**	**x/a**	**y/b**	**z/c**	**U [Å^2]**
V1	4a	0.6760(2)	0.5251(1)	0.5957(3)	0.012(1)
F1	4a	0.3504(7)	0.5023(3)	0.5048(4)	0.016(1)
F2	4a	0.5237(6)	0.6617(3)	0.7331(4)	0.015(1)
F3	4a	0.9701(6)	0.4929(3)	0.7289(4)	0.013(1)
F4	4a	0.8255(6)	0.3445(3)	0.4960(4)	0.013(1)
F5	4a	0.5295(6)	0.3387(3)	0.7218(3)	0.013(1)
O1	4a	0.8128(7)	0.6535(4)	0.4858(5)	0.011(1)
Li1	4a	0.8230(2)	0.3440(2)	0.2974(2)	0.017(3)
Li2	4a	0.1767(6)	0.3346(1)	0.6090(2)	0.017(3)
Li3	4a	0.1900(2)	0.6490(2)	0.3755(2)	0.022(4)
